# Evaluating the diagnostic role of in‐bore magnetic resonance imaging guided prostate biopsy: a single‐centre study

**DOI:** 10.1111/ans.17713

**Published:** 2022-04-28

**Authors:** Marc A. Furrer, Anne Hong, David Wetherell, Stefan B. Heinze, Paul Simkin, Ken Chow, Nathan Lawrentschuk, Homayoun Zargar

**Affiliations:** ^1^ Department of Urology Royal Melbourne Hospital Melbourne Victoria Australia; ^2^ Department of Urology, Inselspital, Bern University Hospital University of Bern Bern Switzerland; ^3^ Department of Radiology Royal Melbourne Hospital Melbourne Victoria Australia; ^4^ Department of Surgery University of Melbourne Melbourne Victoria Australia; ^5^ Department of Urology Western Health Melbourne Victoria Australia

**Keywords:** in‐bore biopsy, MRI‐guided biopsy, multiparametric MRI, prostate cancer

## Abstract

**Background:**

To evaluate the role of in‐bore MRI‐guided biopsy (IB‐MRGB) in the diagnosis of clinically significant prostate cancer (csPCa).

**Methods:**

In this tertiary single centre study, a total of 125 consecutive patients receiving IB‐MRGB over a three‐year period were evaluated, including 73 patients who had prior biopsies and 52 biopsy‐naïve patients. We assessed cancer detection rate of patients according to the degree of suspicion based on mpMRI findings. Histopathological data were reviewed by experienced uropathologists.

**Results:**

The mpMRI was suspicious for PCa (PI‐RADS 4/5) in 77% (96/125) and equivocal (PI‐RADS 3) in 23% (29/125). The detection rate for csPCa was 54.2% (52/96) and 20.7% (6/29) for suspicious lesions (PI‐RADS 4/5) and equivocal lesions (PI‐RADS 3), respectively. In subgroup analysis, patients with previous negative biopsy, overall positive biopsy rate and csPCa detection rate were 48.3% (19/35) and 34.5% (13/35), respectively. In patients on AS, 36/44 (81.8%) and 21/44 (47.8%) had PCa and csPCa respectively. In biopsy‐naïve patients 34/52 (65.4%) and 27/52 (51.92%) had PCa and csPCa respectively. Of the patients on AS, 18/44 (41.6%) upgraded from ISUP 1 to ISUP 2 PCa, and 4/44 (9.1%) upgraded from ISUP 1 to ISUP 3 PCa on IB‐MRGB. A total of 14 Clavien‐Dindo≤2 complications occurred in 14 patients (11.2%) that were directly related to the biopsy. No Clavien‐Dindo≥3 complications occurred.

**Conclusion:**

MRI‐targeted biopsy is suitable for assessment of csPCa. Given the favourable complications profile, its use may be considered in both the initial biopsy and re‐biopsy settings.

## Graphical abstract

In‐bore MRI guided biopsy of the prostate gland provides accurate is safe and feasible method to investigate suspicions of prostate cancer.

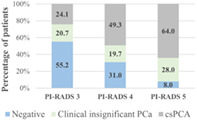

## Introduction

Prostate cancer (PCa) is the second most common cancer amongst men.^1^ The incidence of PCa is increasing with an estimated 1.3 million new cases worldwide in 2018.^1^ The conventional diagnostic pathway for patients with suspected PCa involves serum prostate specific antigen (PSA), digital rectal examination (DRE) and prostate biopsy, either via transrectal ultrasound‐guided (TRUS) or transperineal (TP) approach. Traditionally 12‐core TRUS‐biopsy were performed. However, TRUS‐biopsies demonstrate PCa detection rates are 40–45%,^2^ and underdetection of clinically significant prostate cancer (csPCa), with sensitivity of 48%.^2^ Conversely, estimated overdiagnosis of clinically insignificant PCa range from 1.7% to 46.8%^3^ with standard TRUS‐biopsy. Overtreatment was represented by 27% of men who did not receive any attempted curative treatment in low‐risk prostate cancer.^3^ Therefore, improved methods of evaluating patients with raised PSA and abnormal DRE findings are required. Multiparametric magnetic resonance imaging (mpMRI) prior to prostate biopsy have been increasingly adopted,^4^ and this enables MRI‐guided biopsies.

mpMRI with standardized Prostate Imaging‐Reporting and Data System (PI‐RADS) has improved diagnostic accuracy of PCa, and is incorporated into the pathways for diagnosis of PCa as well as active surveillance (AS).^5^ Several MRI‐targeted guided biopsy techniques have been developed including in‐bore MRI‐guided biopsy (IB‐MRGB). MRI‐targeted biopsy techniques have comparable detection rate of csPCa compared to TRUS‐biopsy but importantly lower rates of detection of insignificant prostate cancer.^6^ The aim of this study was to analyse the role mpMRI and IB‐MRGB for evaluation of csPCa.

## Methods

The present study protocol was approved by the local ethics committee (Office for Research at Melbourne Health, approval number QA 2020014), and the study was conducted in accordance with the principles of the Helsinki Declaration.

In this analysis of a prospectively collected database from a tertiary single centre study, we examined 125 patients who underwent IB‐MRGB between June 2016 and August 2019. Patient details collected include age at IB‐MRGB, serum PSA prior to IB‐MRGB, DRE findings, previous biopsy method (TRUS or TP) and histology.

Patients underwent mpMRI either at our institution or at an external service. The indication for IB‐MRGB was presence of PI‐RADS≥3 in patients on AS, biopsy‐naïve with clinical suspicion of PCa, or those with prior negative biopsy but ongoing suspicion of csPCa. Lesions amenable to biopsy are those within 4 cm of the rectum, limited by the reach of our biopsy needle. Lesions deemed not amendable include anterior lesions, lesions in the extreme apex,^7^ or basal lesions in large volume prostates due to physical inaccessibility.

Our centre utilized only the in‐bore method of biopsies. Two radiologists experienced in prostate imaging and transrectal biopsy technique were involved (SH and PS). Initial mpMRIs were reviewed (i.e., proceduralists were not blinded). The two radiologists performed all IB‐MRGBs in a subsequent session and both utilized the same technique on a 3 T machine. Pre‐procedure enema and oral ciprofloxacin were given on the day of procedure. If the patient had proven extended spectrum beta‐lactamase (ESBL) producing bacteria on previous urine culture, then intravenous ertapenem was also given for prophylaxis. Full details of the biopsy procedure are in Appendix 2.

Patient tolerance of the IB‐MRGB is not formally assessed in this study. However, as patients already had an mpMRI prior to the biopsy, this selects for patients who have sufficient tolerance of the MRI to also tolerate an IB‐MRGB.

Histopathological data were reviewed by experienced uropathologists. Gleason scoring was then verified by a senior uropathologist and followed the recommendations of the 2005 consensus conference of the International Society of Urological Pathology (ISUP). csPCa was defined as ISUP≥2.^8^ Outcomes following IB‐MRGB were collected, including patients who underwent radical prostatectomy (RP) and RP histology, radiation therapy (RT) and AS.

Separate results were collected for patients with high volume ISUP 1. Maximal cancer core length (MCCL) was used as a surrogate measure of tumour volume in the biopsy specimen, and is defined as ≥6 mm of cancer.^9^

Complications (≤90 days) were assessed during the admission and follow‐up at 1 week and 3 months. All patients were either reviewed in clinics or by phone call. For patients undergoing active treatment, complications were assessed at the commencement of this. The definition of sepsis was according to the ‘Third International Consensus Definitions for Sepsis and Septic Shock’.^10^ Complications were classified using the Clavien–Dindo system.^11^

The primary endpoint was csPCa detection. Secondary endpoints included overall PCa detection, positive biopsy stratified by PI‐RADS score and complications.

Descriptive statistics were performed. Statistical significance was calculated using the either the Pearson‐Chi‐squared test or the Fisher's exact test. Statistical analysis was performed using SPSS v. 26 (IBM Corp., Armonk, NY, USA).

## Results

Of 125 patients, 52/125 patients were biopsy naïve, 35/125 patients had prior negative biopsy and 38/125 patients were on AS (Table 1).

**Table 1:** Characteristics of the IB‐MRGB cohort

			IB‐MRGB Only	Previous biopsy	
	Characteristics		(*n* = 52)	(*n* = 73)	*p*
	Median age (IQR)		64.8 (59.3–69.9)	66.2 (60.7–70.4)	0.795
	Pre‐op PSA		6.0 (4.3–7.2)	6.5 (4.9–9.5)	0.051
	Clinical T‐stage	1	34 (65.4%)	50 (68.5%)	0.847
		2	18 (34.6%)	23 (31.5%)	
Previous Biopsy	Method	TP		46 (63.0%)	
		TRUS		27 (37.0%)	
	Gleason grade	Benign		29 (39.7%)	
		ASAP		6 (8.2%)	
		3 + 3		37 (50.7%)	
		3 + 4		1 (1.4%)	
	ISUP grade	Benign/ASAP		35 (47.9%)	
		1		37 (50.7%)	
		2		1 (1.4%)	
	Positive cores			1 (0–2)	
	Cores			20 (12–25)	
MRI	PI‐RADS	3	14 (26.9%)	15 (20.5%)	0.464
	(*n*, %)	4	31 (59.6%)	40 (54.8%)	
		5	7 (13.5%)	18 (24.7%)	
IB‐MRGB results	Positive results grouped by PI‐RADS score (*n*, %)	Overall	34/52 (65.4%)	50/73 (68.5%)	0.698
		csPCa (ISUP≥2)	27/52 (51.9%)	30/73 (41.1%)	0.856
		PI‐RADS 3	5/14 (35.7%)	4/15 (26.7%)	0.452
		PI‐RADS 4	19/31 (61.3%)	17/40 (42.5%)	0.448
		PI‐RADS 5	6/7 (85.7%)	10/18 (55.5%)	0.490
	Cores		5 (4–7)	5 (4–6)	
	Positive cores (median, IQR)		4 (0–4)	3 (0–4)	

Abbreviations: ASAP, atypical small acinar proliferation; MRI, magnetic resonance imaging; PI‐RADS, Prostate Imaging‐Reporting and Data System; IB‐MRGB: in‐bore magnetic resonance imaging guided biopsy; IQR, interquartile range; ISUP, International Society of Urological Pathologist; TP: transperineal, TRUS: Transrectal ultrasound guided.

Median age was 65 years (IQR: 59.0–70.1) and mean serum PSA prior to biopsy was 6.3 ng/dL (IQR: 4.6–8.4). mpMRI was suspicious for PCa (PI‐RADS 4/5) in 96/125 (76.8%) patients and equivocal (PI‐RADS 3) in 29/125 (23.2%) patients.

**Fig. 1:**
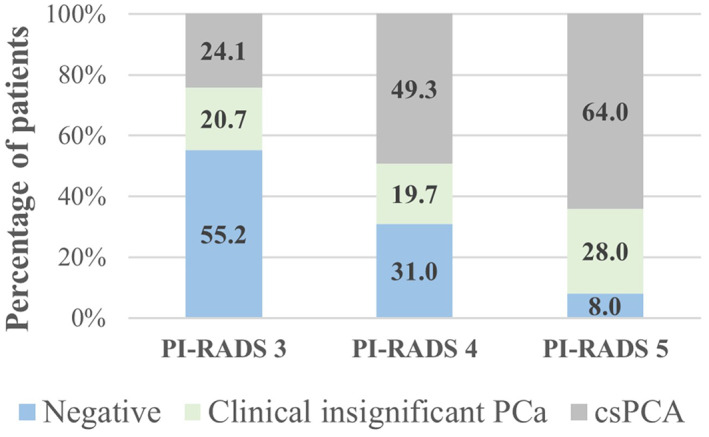
Images demonstrating the IB‐MRGB procedure. (a) Axial T2 images obtained during 45 procedure demonstrating lesion of interest (white arrow), (b) oblique axial view confirming the needle 46 guide position targeting the lesion of interest (white arrow), (c) further confirmation of needle guide 47 position in oblique sagittal view and (d) oblique axial image demonstrating needle stylet passed through 48 the lesion of interest.

The median size of targeted lesions was 11 mm (range: 4–34 mm). PCa was detected in 84/125 (67.2%) of IB‐MRGB. Detection rate of csPCa was 58/84 (69.0%). Detection rate for csPCa in suspicious lesions (PI‐RADS 4/5) was 52/96 (54.1%) and 6/29 (20.7%) for equivocal (PI‐RADS 3) lesions (Fig. 1). In subgroup analysis, patients with previous negative biopsy, overall positive biopsy rate and csPCa detection rate were 14/29 (48.3%) and 10/29 (34.5%) respectively. In AS patients, 36/44 (81.8%) and 21/44 (47.8%) had PCa and csPCa respectively. In biopsy‐naïve patients 34/52 (65.4%) and 27/52 (51.92%) had PCa and csPCa respectively. Of the patients on AS, 18/44 (41.6%) upgraded from ISUP 1 to ISUP 2 PCa, and 4/44 (9.1%) upgraded from ISUP 1 to ISUP 3 PCa on IB‐MRGB.A total of 84 patients had a positive IB‐MRGB of which 39 proceeded with AS. Of 45 patients who had active treatment, 30 underwent RP and 15 underwent RT. Final RP histology was available in all 30 cases and Gleason grade correlated with IB‐MRGB in 17 (56.7%) cases, with 7 (23.3%) upgraded and 6 (20%) downgraded on final histology. The 7 upgraded cases included one case from ISUP 3 to ISUP 5, one case from ISUP 3 to ISUP 4, two cases of ISUP 2 to ISUP 3 and one case from ISUP 1 to ISUP 2. The 6 downgraded cases included 2 cases of ISUP 2 to ISUP 1, 1 case of ISUP 3 to ISUP 1, 2 cases of ISUP 3 to ISUP 2 and 1 case of ISUP 4 to ISUP 3.

Of the patients who had ISUP 1 PCa, 10/27 (37.0%) had MCCL of 6 mm or more. All of these patients underwent AS.

A total of 14 minor complications (Clavien–Dindo ≤2) occurred in 14/125 patients (11.2%): five cases of urinary tract infections (UTI) (two needing intravenous antibiotics, three needing oral antibiotics) (Clavien–Dindo 2), one vasovagal episode needing intravenous fluids (Clavien–Dindo 1), four cases of haematospermia not needing treatment (Clavien–Dindo 1), three cases of macroscopic haematuria including two needing catheterisation and bladder washout (Clavien–Dindo 2) and one not needing intervention (Clavien–Dindo 1) and one episode of urinary retention needing catheterisation. No Clavien–Dindo≥3 complications occurred.

## Discussion

With increasing utility of pre‐biopsy mpMRI,^4^ there is increasing opportunities to target lesions of interest during biopsies. Targeting is performed in a number of ways: cognitive fusion, software fusion, and in‐bore biopsies. mpMRI with subsequent MRI‐guided biopsies showed lower detection rate of low‐risk prostate cancer and improved detection of csPCa when compared to systematic TRUS‐guided biopsies.^12^ Our data is in line with current reports. We found that detection rate of overall PCa for IB‐MRGB is 67.2%, of which and 69.0%% showed csPCa, compared to TRUS‐biopsy, which has a detection rate for overall PCa of 40–45%.^2^

More recently, TP‐biopsies have increasingly replaced TRUS‐biopsies in certain institutions given the favourable infectious complications profile.^13^ Further development of the MRI‐guided biopsy has enabled targeting of biopsies to any lesions of interest. Detection rate using cognitive‐targeted TP‐biopsies is 70% for PCa detection, of which 82% are csPCa.^14^ Using MRI/ultrasound software fusion for TRUS‐biopsy, PCa detection of 62%, of which 71% are csPCa.^14^ Using software fusion in TP‐biopsy, PCa detection rate of 37–56%, of which 68–75% are csPCa.^15^, ^16^ Detection rate in our study is similar to these results. Indeed, the current literature suggest there is no difference between the different types of MRI‐guided biopsies.^17^, ^18^

In our study, we found that of the patients who have previously underwent a biopsy with either negative or insignificant results, 42.4% of those who underwent an IB‐MRGB demonstrated csPCa. This highlights that IB‐MRGB may be particularly useful for repeat biopsies. Comparatively, 28% of patients can avoid a biopsy with negative mpMRI results.^19^ Thus, the pre‐biopsy MRI not only reduces potentially diagnosis of low risk‐disease, but also enabling targeted biopsies to detect what would otherwise be missed malignancies. It is notable that in our subgroup analysis, patients with previous negative biopsy 48.3% had PCa detected. This could be due to the higher representation of anterior lesions in this cohort.^20^ Similarly, in AS patients where 47.8% had csPCa diagnosed, there is high representation of anterior lesions.^21^

We chose the in‐bore technique for our biopsy as it allowed direct visualization of the lesion of interest within the same system. This aimed to reduce sampling error and false negatives, which is particularly significant for targeting smaller lesions as the needle can be visualized piercing the target lesion (Appendix 2). A more recent comparison between MRI‐US fusion versus MRI in‐bore methods of biopsy suggests that PI‐RADS score was a significant predictor of positive targeted cores, whereas the different methods of biopsy showed comparable ISUP grade results.^22^

We observed that higher rates of csPCa are associated with higher PI‐RADSv2 rating, a finding seen in prior studies.^19^ While the urological community would agree that PI‐RADS 4 and 5 lesions are high risk, PI‐RADS 3 lesions can be a diagnostic challenge.^23^ We observed 20.7% of PI‐RADS 3 lesions demonstrated csPCa. This contrasted with some recent work. Liddell *et al*. found 4.3% of PI‐RADS 3 lesions contained csPCa according to Epstein's criteria.^23^ Accordingly, there have been suggestions to consider follow‐up rather than biopsy.^24^ Our results are different possibly owing to the recent upgrade in PI‐RADS system to version 2. As such, we cannot recommend follow‐up over biopsy of PI‐RADS 3 lesions.

As expected, detection rate for both PCa and csPCa increased with PI‐RADS score, where 67.6% of PI‐RADS 4 lesion showed PCa and 92% of PI‐RADS 5 lesions showed PCa. csPCa detection rate for PI‐RADS 5 lesions was 64.0%. This is low comparing to a recent review where 72% of PI‐RADS 5 lesions represented csPCa.^25^ Our results may be due to the 73 patients with prior negative biopsies or on AS. This subset had 18 patients with PI‐RADS 5 lesions, of which 10 had csPCa, which is similar to reported data.^26^ Furthermore, Hambrock *et al*. also demonstrated 31 of 46 tumours were anterior in a series of men with repeated negative biopsies,^27^ suggesting that anterior lesions may account for missed diagnosis in men with previous biopsies. If we only assessed biopsy‐naïve men with PI‐RADS 5 lesions, 85% (6 out of 7) demonstrate csPCa which is in line with expectations.

Several prior studies examined the concordance between systematic prostate biopsy Gleason grades and RP Gleason grades. Specifically, De Luca *et al*. reported rates of upgrading on systematic biopsies were 38.8% versus 16.7% for targeted biopsies.^28^ Within our study, 23 men underwent RP with 4/125 (17.4%) cases being upgraded, is in concordance with De Luca's results, suggesting higher accuracy of diagnosis with MRI‐guided biopsy techniques. Spearman's coefficient was statistically significantly, further reinforcing this positive correlation.

Given that IB‐MRGB relies on the mpMRI, our study relies on the accuracy of mpMRI to detect suspicious lesions. Le *et al*. found that mpMRI had a sensitivity of 72% for tumours with ISUP≥2^29^ and the PROMIS trial demonstrated that mpMRI was significantly more sensitive than TRUS‐biopsy in detecting ISUP≥2 tumours.^2^ However, Bratan *et al*. demonstrated a false positive rate of 40–42%,^30^ and our results could be confounded by this.

We performed IB‐MRGB via the transrectal route. This was chosen as patients required only sedation and is also the route of IB‐MRGB with the most experience.^31^ A shortcoming of TRUS‐biopsies is the higher rate of infection complications compared to TP‐biopsies,^32^ with rates of hospital readmission from TRUS‐biopsy‐related infections ranging from 1.1% to 4.1%.^33^, ^34^, ^35^ We aim to reduce these risks by taking 3–4 passes of the needle, less than that of a systematic biopsy.

Our antibiotic regimen included oral ciprofloxacin given on the day of procedure, with additional ertapenam if there was proof of ESBL bacterium. We observed five (4.0%) cases of UTIs, with two patients requiring intravenous antibiotics. This was comparable to the rates of infectious complications from TRUS‐biopsies.^36^Reports of more extensive antibiotic regimes used include intravenous antibiotic peri‐biopsy or taking oral fluoroquinolone pre‐ and post‐biopsy have reported lower sepsis rates.^37^, ^38^ Furthermore, TP‐biopsies with single dose ceftriaxone prophylaxis report infections rates of 0.01%.^39^ This is much improved from our complications rates and prompt us to re‐evaluate our antibiotic regime.

Vast majority of studies on IB‐MRGB have been done via the transrectal route, with one study examining TP‐biopsy and one study examining transgluteal biopsy.^31^ Given the rise in infections post TRUS‐biopsies, rise in antibiotic resistant organisms,^40^ and the reduced complication rates of TP‐biopsy,^32^ these alternate routes of IB‐MRGB require further evaluation. In the interim, we argue that transrectal IB‐MRGB with more extensive prophylactic antibiotic regime has a role in diagnosis of csPCa.

We observed 2.4% patients experiencing haematuria, which is lower than the 50% reported risk.^36^ Furthermore, 0.8% of our patients experienced urinary retention not due to clots and falls within the range of 0.2–2.6% reported risk.^36^ Finally, 3.2% patients experienced haematospermia but none required treatment, which is also much lower than the 50% reported.^36^ Overall, our non‐infectious complication rates are lower than those reported in literature for prostate biopsy, inferring that IB‐MRGB is a safer technique in this respect.

Our study was not without limitations, and some have been discussed above. Given our IB‐MRGB is via the transrectal route, it is also not suitable for lesions in inaccessible areas of the prostate. We relied on mpMRI technology, which has inherent shortcomings discussed above.

Another limitation of this study was selection bias. It was designed to assess the detection of csPCa in patients who have undergone IB‐MRGB, hence only patients who underwent IB‐MRGB were included. It also comprised a small cohort of patients.

Finally, the diagnostic mpMRIs are not all performed in our institution and as such, we were not able to control this process. We aim to reduce the potential confounding by reviewing PI‐RADS 3 or above lesions at the MDTM where there are three experienced radiologists present to form a consensus. Furthermore, using external mpMRIs are a closer representation of real‐world scenarios, thus improves applicability of our data. These shortcomings notwithstanding, our study add to the growing body of literature supporting the judicious use of mpMRI and IB‐MRGB in selected patients.

## Conclusion

Our study contributes to evidence that mpMRI and MRI‐targeted biopsy provide valuable additions to the diagnostic evaluation of csPCa. With changes to our antibiotic regime and potential improvements to our infectious complications profile it may be appropriate as the biopsy technique of choice prior to systematic biopsy in Australia. Despite the limitations of our study, it supports the use of mpMRI and MRI‐targeted biopsy as part of the diagnostic algorithm for csPCa.

## Conflict of interest

None declared.

## Previous communications

Preliminary results of this study was presented as a poster at the 40th Congress of the Société Internationale d'Urologie held on 10–11th October 2020.

## Author contributions

**Marc Furrer:**Data curation; formal analysis; writing – review and editing. **Anne Hong:** Data curation; formal analysis; writing – original draft. **David Wetherell:** Data curation; formal analysis; methodology; project administration. **Stefan Heinze:** Investigation; writing – review and editing. **Paul Simkin:** Investigation; writing – review and editing. **Ken Chow:** Formal analysis. **Nathan Lawrentschuk:** Writing – review and editing. **Homayoun Zargar:** Conceptualization; investigation; project administration; supervision; writing – review and editing.

## Supporting information


**Appendix S1:**Supporting Information
