# Supplementary material for: Evaluating the diagnostic role of in‐bore magnetic resonance imaging guided prostate biopsy: a single‐centre study
Source: ANZ J Surg. 2022 Apr 28;92(6):1486–91. doi: 10.1111/ans.17713 (PMC9324140; doi:10.1111/ans.17713)
Supplement: Supplementary file 1 — Appendix S1:Supporting Information [file ANS-92-1486-s001.docx]

**Appendix 1: Multiparametric Magnetic Resonance Imaging Protocol and Reporting**

Multiparametric magnetic resonance imaging (mpMRI) were performed either at 1.5T or 3T, either within our institution or externally. Should the patient have the mpMRI within our institution, a 3T MRI with a pelvic coil is used unless contraindicated. The images obtained were as follows:

- T2 images are 3 mm contiguous slices, axial, sagittal and coronal planes, with in plane resolution of 0.7 mm x 0.625 mm.
- Diffusion weighted images (DWI) were taken in b-values of 50, 400 and 1000, with the b-value of 1600 being calculated. These have an in-plane resolution of 2.3 mm x 1.8 mm.
- Dynamic contrast-enhanced images were acquired with in plane resolution 2.0 mm x 1.6 mm and a temporal resolution of 7 seconds. 10 time points were acquired with no processing afterwards.

Apparent diffusion coefficient and high b-value DWI images were used qualitatively. An example of mpMRI is seen below in **Figure 1**. All mpMRI scans were reported by the on-duty radiologist. Within our institution, all scan are double reported independently. Any suspicious lesions were assigned a PI-RADSv2 score from 1 (low) to 5 (high) according to the likelihood of cs-PCa being present ^1^. Reporting was based on the template from the PI-RADSv2 guidelines, including a diagram. Any mpMRIs with PI-RADS≥3 were reviewed again at a multidisciplinary team meeting (MDTM) where three radiologists experienced in the reporting of mpMRI images were present, along with the entire urology unit. Experience levels of the three radiologists are 20 years, 7 years, and 5 years. We do not have this information for radiologists who are external to our institution.

Figure 1 – diagnostic MRI images in the axial plane of a lesion of interest in the right lateral peripheral zone (arrow) in (A) axial T2 weighting, (B) axial apparent diffusion coefficient weighting and (C) high B-value diffusion weighted


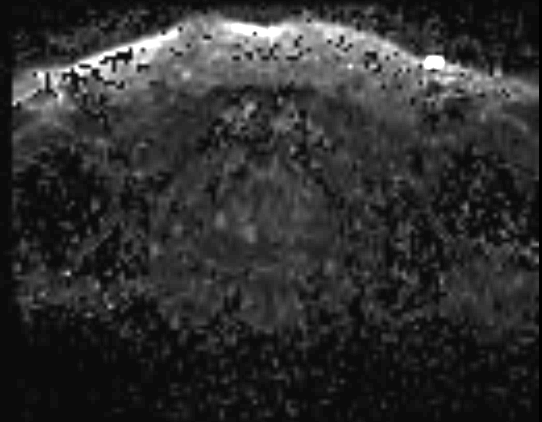

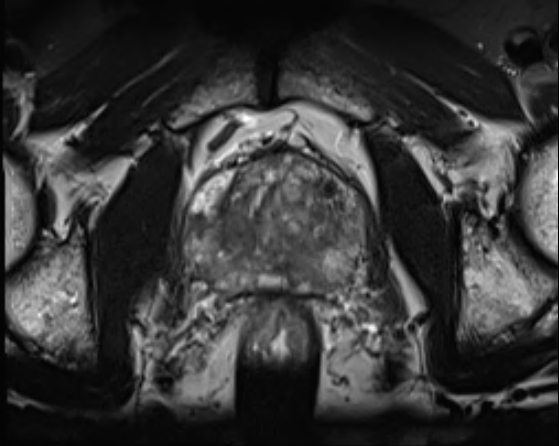

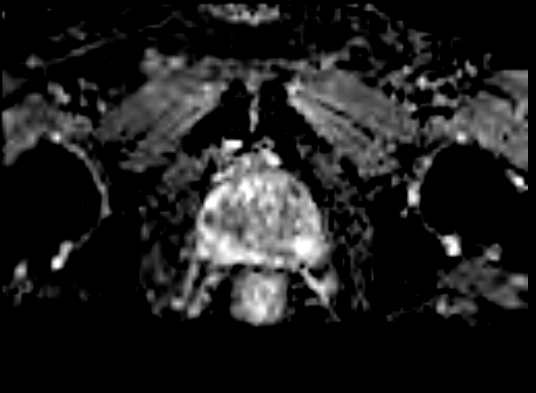


**A.**

**C.**

**B.**

**Appendix 2: In-bore MRI guided biopsy procedure**

Pre-procedure enema and oral ciprofloxacin were given routinely on the day of procedure. If the patient had proven extended spectrum beta-lactamase (ESBL) producing bacteria on previous urine culture that is resistant to ciprofloxacin, then intravenous ertapenem was given for prophylaxis. Details of the procedure are given in **table 1** below. In summary, the patients were positioned prone on the MRI scanner. Intravenous sedation using fentanyl was administered by the radiology department nursing staff. Specimens of the lesion(s) were obtained (**Figure 2**) using clampstand and needle sleeve, and 18-gauge MRI compatible core biopsy needle (Invivo, Gainsville, FL, USA). 3-4 biopsies ^2, 3^ were obtained from each target lesion to ensure significant disease was not missed ^4, 5^. Accuracy of the target is confirmed on repeat MRI images demonstrating the needle stylet position relative to the lesion (**Figure 2**). Logistically, 1-hour bookings sessions are made for each biopsy leaving allowances for patient transfer, set up time, sedation and post procedure disinfection. There was no data formally collected for the patients in-bore duration, however this is estimated at 20-45 minutes by the proceduralists.

Table 1 – Procedural steps for performing IB-MRGB

| **A. Pre-procedural preparation** |
| --- |
| 1. Patient is fasting for at least 6 hours |
| 1. Images of the prior diagnostic study were reviewed (Figure 1) |
| 1. Pre-procedure antibiotics and an enema given |
| **B. Procedure** |
| 1. Patient positioned prone in the magnet |
| 1. Preliminary T2 images to confirm target lesion (Figure 2)   If target lesion is not visible on T2 images, DWI/ADC images are acquired to localize lesion, with cross registration to T2 images. |
| 1. Light conscious sedation given |
| 1. Insertion of transrectal needle guide with local anaesthetic lubricant |
| 1. Attach needle guide to gantry approximating the position of the lesion |
| 1. Oblique axial and oblique sagittal images in T2 weighting obtained along the needle guide to confirm position of guide relative to the lesion (Figure 2) |
| 1. Adjust needle guide as needed |
| 1. Repeat steps 6 and 7 as needed until needle guide is appropriately positioned |
| 1. Measure distance of lesion from needle guide |
| 1. Insert biopsy needle to appropriate depth, deploy stylet |
| 1. Repeat acquisition of MRI images per step 6 to confirm stylet through lesion (Figure 2) |
| 1. Obtain 3-4 biopsy specimens |
| 1. Remove needle guide |
| 1. Patient transferred to recovery area |

Figure 2 – images demonstrating the IB-MRGB procedure. A) axial T2 images obtained during procedure demonstrating lesion of interest (white arrow), B) oblique axial view confirming the needle guide position targeting the lesion of interest (white arrow), C) further confirmation of needle guide position in oblique sagittal view, D) oblique axial image demonstrating needle stylet passed through the lesion of interest.


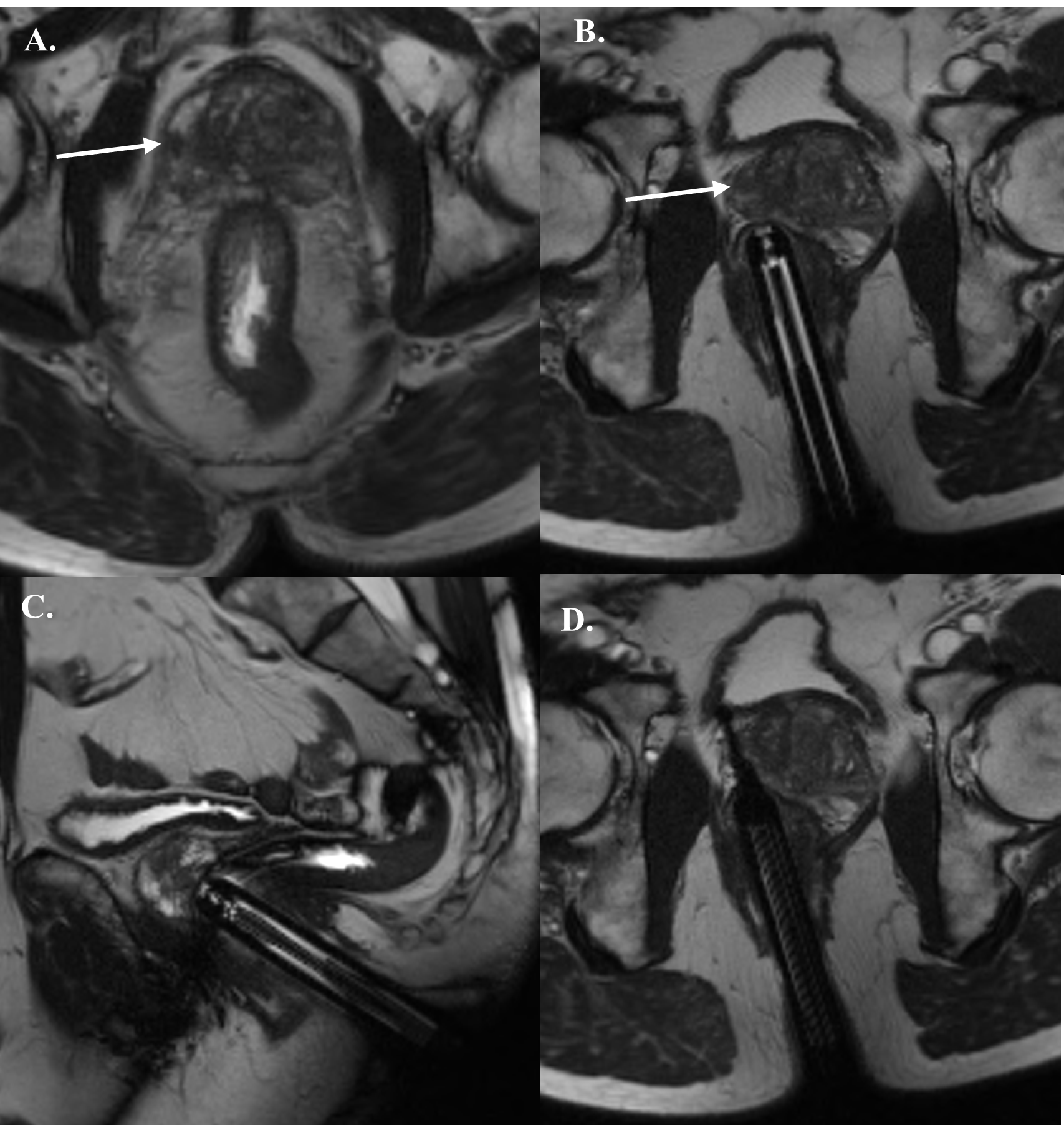


**References:**

[1] Weinreb JC, Barentsz JO, Choyke PL, et al. PI-RADS Prostate Imaging - Reporting and Data System: 2015, Version 2. *Eur Urol*. 2016; 69:16–40.

[2] Song G, Ruan M, Wang H, et al. How Many Targeted Biopsy Cores are Needed for Clinically Significant Prostate Cancer Detection during Transperineal Magnetic Resonance Imaging Ultrasound Fusion Biopsy? *J Urol*. 2020; 204:1202-8.

[3] Sonmez G, Demirtas T, Tombul ST, Ozturk F, Demirtas A. What is the ideal number of biopsy cores per lesion in targeted prostate biopsy? *Prostate International*. 2020; 8:112-5.

[4] Kenigsberg AP, Renson A, Rosenkrantz AB, et al. Optimizing the Number of Cores Targeted During Prostate Magnetic Resonance Imaging Fusion Target Biopsy. *Eur Urol Oncol*. 2018; 1:418-25.

[5] Lu A, Ghabili K, Nguyen K, Leapman M, Sprenkle P. How many cores are needed to detect clinically significant prostate cancer on targeted MRI-ultrasound fusion biopsy? *Journal of Clinical Oncology*. 2018; 36:134-.
